# Non-Heading Chinese Cabbage Database: An Open-Access Platform for the Genomics of *Brassica campestris* (syn. *Brassica rapa*) ssp. *chinensis*

**DOI:** 10.3390/plants11081005

**Published:** 2022-04-07

**Authors:** Zhidong Li, Ying Li, Tongkun Liu, Changwei Zhang, Dong Xiao, Xilin Hou

**Affiliations:** State Key Laboratory of Crop Genetics & Germplasm Enhancement, Key Laboratory of Biology and Genetic Improvement of Horticultural Crops (East China), Ministry of Agriculture and Rural Affairs of the P. R. China, Engineering Research Center of Germplasm Enhancement and Utilization of Horticultural Crop, Ministry of Education of the P. R. China, College of Horticulture, Nanjing Agricultural University, Nanjing Suman Plasma Engineering Research Institute, Nanjing 210095, China; 2018204017@stu.njau.edu.cn (Z.L.); yingli@njau.edu.cn (Y.L.); liutk@njau.edu.cn (T.L.); changweizh@njau.edu.cn (C.Z.); dong.xiao@njau.edu.cn (D.X.)

**Keywords:** *Brassica campestris*, non-heading Chinese cabbage, whole-genome sequence, genome database

## Abstract

The availability of a high-quality genome sequence of *Brassica campestris* ssp. *chinensis* NHCC001 has paved the way for deep mining of genome data. We used the *B. campestris* NHCC001 draft genome to develop a comprehensive database, known as the non-heading Chinese cabbage database, which provides access to the *B. campestris* NHCC001 genome data. The database provides 127,347 SSR, from which 382,041 pairs of primers were designed. NHCCDB contains information on 105,360 genes, which were further classified into 63 transcription factor families. Furthermore, NHCCDB provides eight kinds of tools for biological or sequencing data analyses, including sequence alignment tools, functional genomics tools, comparative genomics tools, motif analysis tools, genome browser, primer design, and SSR analysis tools. In addition, eight kinds of graphs, including a box plot, Venn diagram, corrplot, Q-Q plot, Manhattan plot, seqLogo, volcano plot, and a heatmap, can be generated rapidly using NHCCDB. We have incorporated a search system for efficient mining of transcription factors and genes, along with an embedded data submit function in NHCCDB. We believe that the NHCCDB database will be a useful platform for non-heading Chinese cabbage research and breeding.

## 1. Introduction

*Brassica campestris* (AA genome, 2n = 2x = 20), which is often used as a synonym for *Brassica rapa*, belongs to the family of Brassicaceae. The family Brassicaceae includes several commercially important oilseeds, vegetables, and medicinal crops, which can serve as good models for scientific research. These include *Brassica nigra* (BB, 2n = 2x = 16), *Brassica oleracea* (CC, 2n = 2x = 18), *Brassica juncea* (AABB, 2n = 4x = 36), *Brassica napus* (AACC, 2n = 4x = 38), and *Brassica carinata* (BBCC, 2n = 4x = 34) [1]. *Brassica campestris* is a highly morphologically diverse species that includes non-heading Chinese cabbage (subsp. *chinensis*), heading Chinese cabbage (subsp. *pekinensis*), turnip (subsp. *rapa*), oil types (subsp. *oleifera*), rapini (subsp. *sylvestris*), and yellow sarson types (subsp. *trilocularis*) [2].

The genetic system of the Brassicaceae family, particularly of the U’s triangle model [1], provides a good opportunity to study interspecies hybridization and genome evolution in polyploids. The heading Chinese cabbage (*B*. *rapa* Chiifu) and turnip (*B*. *rapa* Z1) genomes provide a rich resource for genetic and comparative genomics analysis of *B*. *rapa* [3,4,5,6,7]. Pak choi, a non-heading form of Chinese cabbage, is a popular vegetable in China and other East Asian regions, known for its nutritious and succulent stalks and leaves. Recently, we presented a chromosome-level assembly of *Brassica campestris* (syn. *Brassica rapa*) ssp. *chinensis* “NHCC001” [8] (“Suzhouqing”, green petiole type pak choi) produced by PacBio technology, “ZYCX” (a cultivar of pak-choi) genomes [9] are being sequenced at the same time, which provides a novel resource for non-heading Chinese cabbage genomic research. However, no database is available for non-heading Chinese cabbage, limiting the research. In this context, it was necessary to build an integrative functional genomic data analysis platform for non-heading Chinese cabbage.

Based on the *B. campestris* “NHCC001” reference genome [8] and a collection of 16 genomic data of seven Brassicaceae species (Table S1), we present a functional genomic database enabled with a user-friendly web interface for non-heading Chinese cabbage, called “Non-heading Chinese Cabbage Database” (NHCCDB, http://tbir.njau.edu.cn/NhCCDbHubs/index.jsp; accessed on 11 January 2022). To allow scientists to efficiently utilize these genomic datasets, we appropriately mined, analyzed, and stored the genomic data in the NHCCDB. Several bioinformatics tools have been embedded in NHCCDB, allowing the users to search, download, analyze, and visualize datasets. We believe that the NHCCDB database will be a useful platform for non-heading Chinese cabbage research and breeding.

## 2. Construction and Content

### 2.1. Genome Sequences and Annotation Resources

Recently, we presented a chromosome-level assembly of Brassica campestris ssp. chinensis “NHCC001” produced by PacBio technology [8]. Based on the *B. campestris* reference genome and annotation resources, we collected 16 released genomic data of seven Brassicaceae species (Table S1). Next, we built the NHCCDB database, from where all genomic data can be easily downloaded.

### 2.2. Transcription Factor Family Identification

Transcription factors (TFs) are key regulators of plant development and several abiotic stresses. The protein sequences collected in the NHCCDB were further compared using the Pfam database (http://pfam.xfam.org/; accessed on 15 January 2020) and the Pfam_Scan.pl program (default cutoff for Pfam-A searches of PfamScan) [10]. From the whole-genome sequences of 17 datasets belonging to seven Brassicaceae species, 105,360 genes were identified and classified into 63 TF families according to the criteria of the Coriander Genomics Database [11].

### 2.3. Simple Sequence Repeat Finder and Primer Design

Simple sequence repeat (SSR) is a commonly used genetic marker in plant genetics and breeding studies. The Microsoft Intelligent Security Association (MISA) program implemented in the MISA [12] database (https://webblast.ipk-gatersleben.de/misa/; accessed on 11 January 2021) was used to identify the SSRs in the coding sequences collected in NHCCDB. Next, we analyzed the *B. campestris* NHCC001 genome data, and from 4771 SSRs, 14,313 pairs of primers were designed. In total, NHCCDB contains 127,347 SSRs, from which 382,041 pairs of primers were designed.

### 2.4. Syntenic Regions

To study the evolutionary relationships and species divergence, syntenic regions between *B. campestris* NHCC001 and *A. thaliana* were identified using the MCscanX software [13]. These can be downloaded and browsed in NHCCDB. First, the BLASTP program with an E-value cutoff of 1 × 10^−5^ was used to identify homologous genes pairs. Next, the MCScanX software, using the parameters k = 50, s = 5, and m = 25, was used to identify the syntenic regions [13]. In total, 2610 syntenic regions, including 53,440 gene pairs, were classified between *B. campestris* NHCC001 and *A. thaliana*.

### 2.5. Literature Collection

NHCCDB aims to provide a public literature repository on the research on plants of *Brassica campestris* ssp. *chinensis*. Currently, the NHCCDB literature module includes 226 papers/books about *Brassica campestris* ssp. *chinensis*, which were obtained from PubMed (https://pubmed.ncbi.nlm.nih.gov/; accessed on 11 January 2022) using NCBI’s E-utilities function [14].

### 2.6. Biological Plot Platform Construction

Data visualization is an important part of bioinformatics analysis. We developed a user-friendly biological plot tool platform “Bioplot”, which supports eight types of biological plotting, including a box plot, Venn diagram, corrplot, Q-Q plot, Manhattan plot, seqLogo, heatmaps, and a volcano plot.

### 2.7. Data Integration and Website Construction

**Figure 1:**
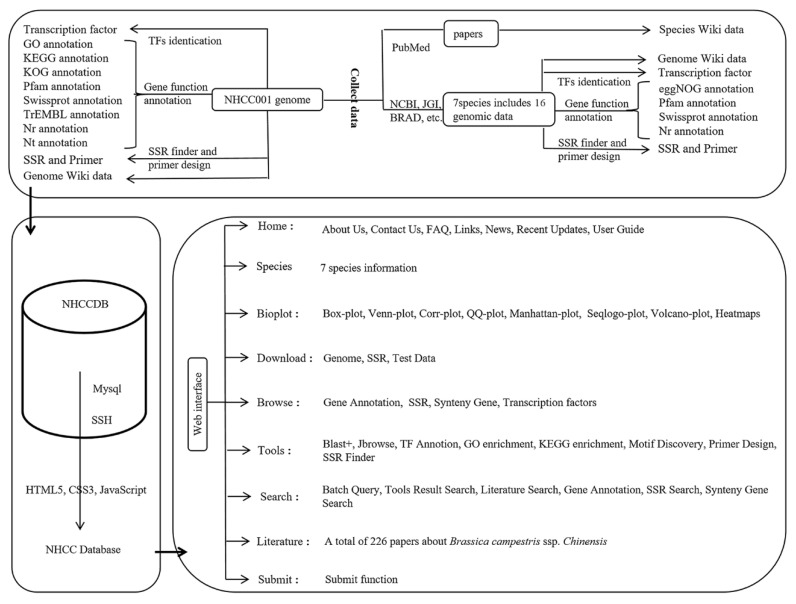
Flow diagram showing the designing and construction of NHCCDB.

NHCCDB was created by integrating a variety of bioinformatics programs on the Linux platform. NHCCDB facilitates the scientific study of genomic data of *Brassica campestris* ssp. *chinensis*. This website was set up on our group’s IBM blade server and used Apache Tomcat as a web server. The collected data were processed using Perl, Python, R scripts, and various bioinformatics tools. All datasets were integrated into the MySQL database. HTML5, JavaServer Pages, CSS3, and jQuery were used in front-end development. ECharts was used to generate dynamic charts. The SSH framework was used to provide query support from the backend of the database (Figure 1).

## 3. Utility and Discussion

### 3.1. A Brief Introduction to the NHCCDB

NHCCDB provides a user-friendly interface to facilitate the retrieval of information. The NHCCDB structure consists of nine main modules: Home, Species, Bioplot, Download, Browse, Tools, Search, Literature, and Submit. From those modules, users can easily perform a data search, downloading, analysis, and visualization of the *B. campestris* NHCC001 and its relative genomic data available in NHCCDB. Therefore, NHCCDB facilitates studies on molecular breeding and comparative and functional genomics research of non-heading Chinese cabbage.

### 3.2. Species Information

**Figure 2:**
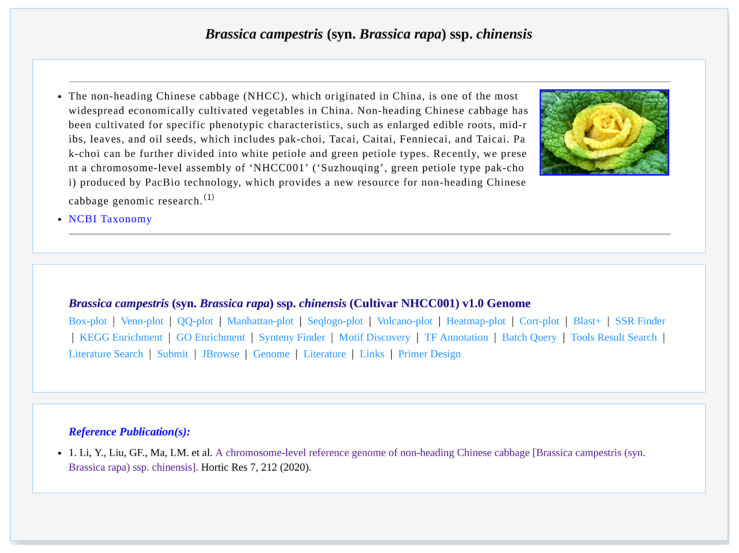
Screenshot of a species page for *Brassica campestris* (syn. *Brassica rapa*) ssp. *chinensis*.

NHCCDB organizes the available deep-sequencing genomic datasets of *B. campestris* and its relative species, manually curated from 83 publications in modules of “Species”, where each species corresponds to a wiki page. Each species wiki page includes the following divisions: the basic description of the species, genomic data, and 24 kinds of data analysis functions and reference(s) (Figure 2). In particular, the content of each genomic wiki page is structured into multiple sections, namely 24 kinds of bioinformatics tools, species overview, assembly data, and reference(s) (Figure S1). Users can easily find information about the desired species in the species module.

### 3.3. Browsing Gene Function Annotation and Simple Sequence Repeat

**Figure 3:**
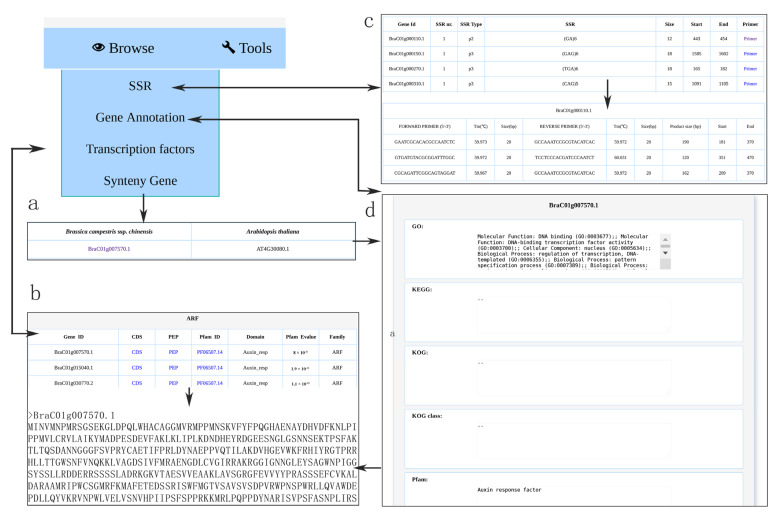
Browse module. (**a**) The syntenic gene in *B. campestris* NHCC001 and *Arabidopsis thaliana*. (**b**). *Brassica campestris* ssp. *chinensis* NHCC001 ARF (Auxin response factor) family. (**c**) Browsing of the SSR dataset of *B. campestris* NHCC001. (**d**) Gene function annotation datasets.

To facilitate the use of the gene annotation resources, the “Gene Annotation” tool was embedded in the NHCCDB database. This functional unit is contained in the “Browse” module. Moreover, it has a user-friendly graphic interface to help users browse the gene function annotation datasets of *B. campestris* NHCC001 (Figure 3d). Next, we analyzed the *B. campestris* NHCC001 genome data and from 4771 SSRs, 14,313 pairs of primers were designed. The “SSR” tool was embedded in the NHCCDB database to help users browse and access the SSR dataset of *B. campestris* NHCC001 (Figure 3c).

### 3.4. Browsing Syntenic Genes and Transcription Factor

To better understand the collinear relationship between species, the syntenic gene in *B. campestris* NHCC001 and *A. thaliana* can be browsed and accessed using the “Syntenic gene” function embedded in the NHCCDB. In total, 2610 syntenic regions, including 53,440 gene pairs, were classified between *B. campestris* NHCC001 and *A. thaliana*. These data can be freely accessed and browsed (Figure 3a).

The “transcription factor” tool contains information on 105,360 genes, further classified into 63 TF families from the whole genome sequences of 17 datasets belonging to seven Brassicaceae species. The users only need to select the species and TF family, and the results page will appear when the users submit the task. On the result page, users can browse and download the coding sequences and protein sequences. In addition, a TF introduction link is provided on the result page, which will redirect the users to the Pfam database (http://pfam.xfam.org/; accessed on 11 January 2022). Here, we use the ARF (Auxin response factor) family as an example to show the TF browse results (Figure 3b).

### 3.5. Sequence Alignment Tools

This database also offers a homology search tool with a user-friendly graphic interface, “Blast+”, which was embedded in the NHCCDB using the BLAST 2.9.1+ program [15]. Users only need to supply a nucleic acid or amino acid sequence in the FASTA format by uploading or directly pasting it into the search box against the available databases, selecting certain values of certain parameters, and the results page will be displayed once the users submit the task.

### 3.6. Functional Genomics Tools

**Figure 4:**
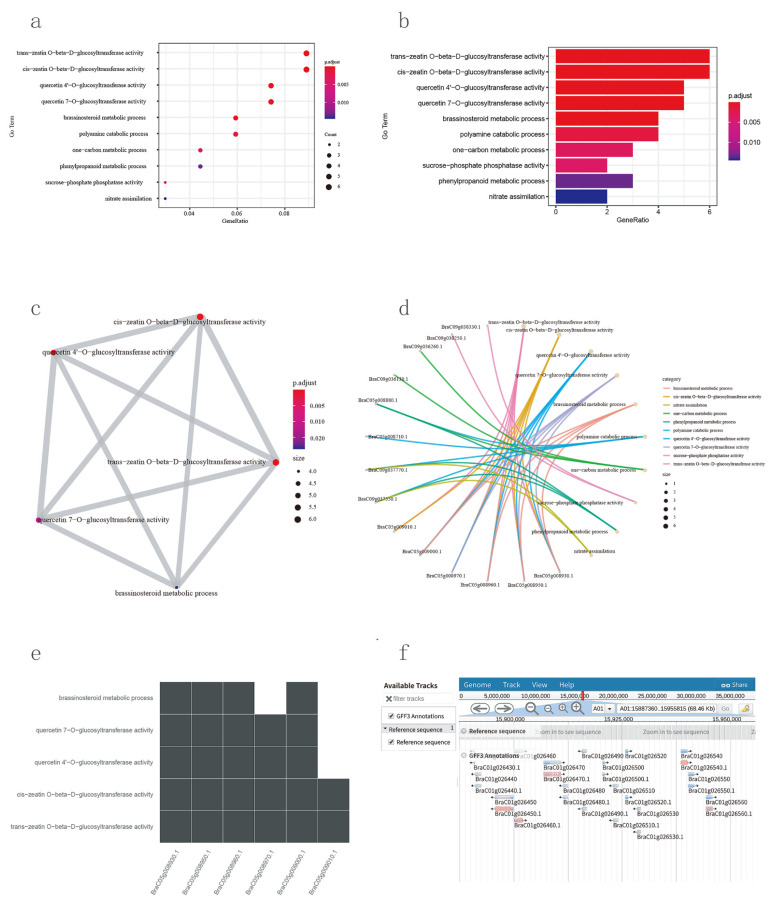
GO and KEGG enrichment and JBrowse tools in NHCCDB. (**a**) Visualization of GO and KEGG enrichment results with bubble charts. (**b**) Visualization of GO and KEGG enrichment results with bar charts. (**c**) Visualization of GO and KEGG enrichment results with emapplots. (**d**) Visualization of GO and KEGG enrichment results with network planning. (**e**) Visualization of GO and KEGG enrichment results with heatmaps. (**f**) Genome browser view of processed genome data.

Three types of functional genomics tools, namely, TF annotation, GO enrichment, and KEGG enrichment, were developed to perform Pfam, GO, and KEGG functional annotation analyses. Additionally, GO and KEGG enrichment results can be visualized in the form of five different plots, such as a bar plot, dot plot, centplot, emapplot, and a heat plot (Figure 4). Users can adjust visualization parameters according to the enrichment results. Furthermore, the browse and download enrichment result functions are provided on the results page.

### 3.7. Primer Design and SSR Finder

Research on gene function is an indispensable component of the molecular plant breeding program. Designing a pair of suitable primers is fundamental to gene cloning. The Primer 3.0 program (https://sourceforge.net/projects/primer3/; accessed on 11 January 2022) [16] was embedded in NHCCDB with a user-friendly graphic interface to design primers of the given genomic sequences. Nucleic acid sequences can be directly submitted by simply pasting the sequence to the frame. The primer design results will be generated when the users provide values for a few parameters and submit the task.

The SSR Finder function is used to identify the SSR in a given genome sequence and design three pairs of primers for the sequence. Pasting nucleic acid sequences or uploading the sequences in the FASTA format can be completed to submit the sequence data. SSR analysis and the sequence of the designed primers will be provided once the users submit the task. On the results page, users can browse and download the analysis results.

### 3.8. JBrowse and Motif Analysis Tool

In NHCCDB, we have incorporated a genome browser (JBrowse), developed using HTML5 and JavaScript [17]. JBrowse can be used to browse and view the genome sequences and gene modules. Presently, this function supports 17 genome datasets. JBrowse supports visualization scaling and smooth transition from the genome level to nucleotide base level. Furthermore, JBrowse supports the query of the genomic sequences at the individual chromosome level. Users can click on gene modules to view gene information such as names, positions, types, lengths, and sequences. This function also supports downloading gene sequences (Figure 4f).

Motif Discovery, developed using the MEME (http://meme-suite.org; accessed on 11 January 2022) program [18], was installed in the NHCCDB to help users perform motif analysis. Users need to upload the sequence data, provide the values for selected parameters, and on submitting the task, the results page is displayed. The analysis result can be browsed and downloaded from the link provided on the results interface.

### 3.9. Biological Plot Platform

**Figure 5:**
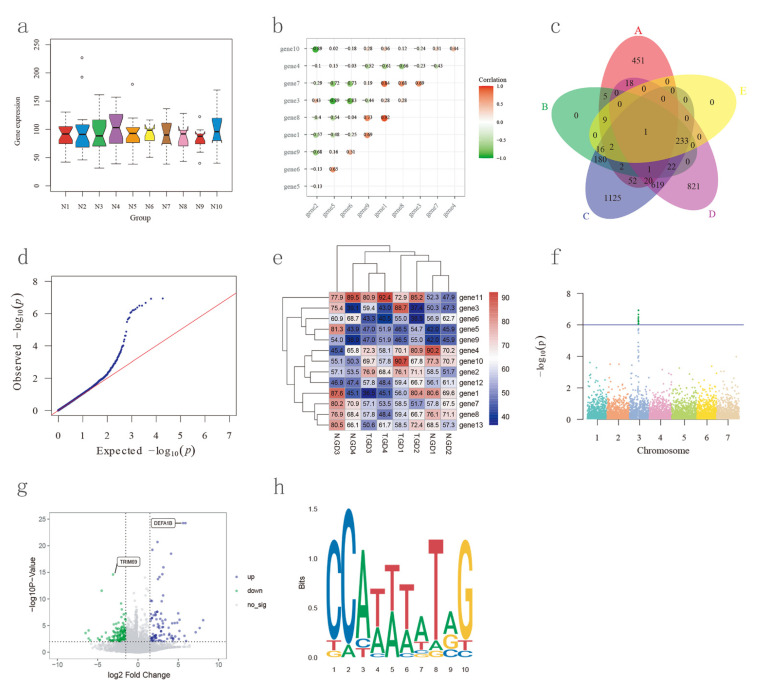
A total of eight kinds of graphs can be generated using NHCCDB. (**a**) Box plot. (**b**) Corrplot. (**c**) Venn diagram. (**d**) Q-Q plot. (**e**) Heatmap. (**f**) Manhattan plot. (**g**) Volcano plot. (**h**) SeqLogo.

In the “Bioplot module”, we developed a user-friendly biological plot tool platform Bioplot. A total of eight kinds of graphs can be rapidly generated using NHCCDB, including a box plot, Venn diagram, corrplot, Q-Q plot, Manhattan plot, seqLogo, heatmap, and a volcano plot (Figure 5). In addition, we provide instruction files in every plot tool interface. The users only need to upload the data, select values for a few parameters, and the plotting results page, allowing the users to browse and download the plotting results, will be displayed once the users submit the task.

### 3.10. Search

A range of search tools has been embedded in the NHCCDB database, including “Batch Query”, “Tools Results Search”, “Literature Search”, “Gene Annotation Search”, “SSR Search”, and “Synteny Gene Search”. These tools can be used to search the gene and protein sequences, tools analysis results, gene function annotation, SSR, synteny genes, and the literature.

**Figure 6:**
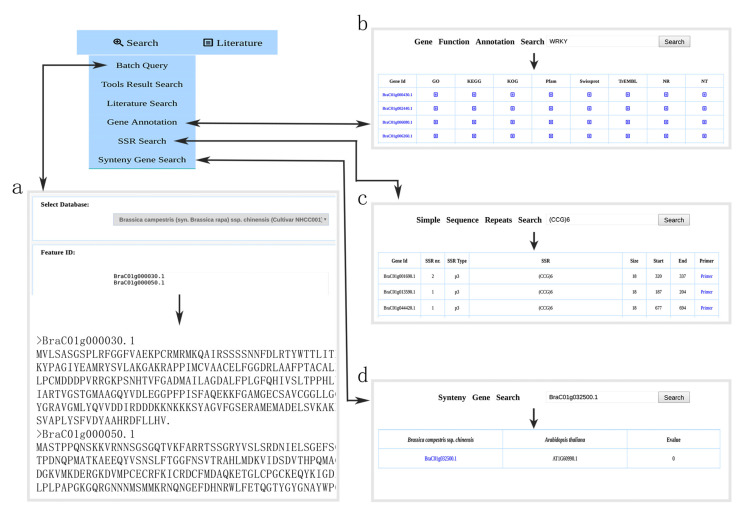
Search module. (**a**) Batch Query tool search results. (**b**) *B. campestris* NHCC001 gene function annotation search result. (**c**) Using SSR (CCG)6 as an example to show SSR search results. (**d**) Syntenic genes search results.

In NHCCDB, scientists working on the Brassicaceae species can find the desired genes or homologous genes in the genomes of the plants of the Brassicaceae family using Blast+ tools. According to the location of the gene, users can locate the desired gene in the genome using the JBrowse tool. According to the BLAST+ and JBrowse analysis results, users can upload the gene identifier file or they can simply paste the gene identifier list into the Batch Query Tool interface. At present, the dataset of the batch query tool includes 17 genomic datasets. The users need to select the dataset of batch query, and the results page will be displayed after submitting the task. We used *B. campestris* NHCC001 genes *BraC01g000030.1* and *BraC01g000050.1* as an example to show the “Batch Query” tool search results (Figure 6a).

Numerous bioinformatics analysis functions were embedded in NHCCDB, which can be used to find similarities and synteny among various gene sequences, perform gene function annotation and motif analysis, identify SSRs, visualize the genome, and design the primers. The tools analysis results are displayed on the results page. Users can browse and download the results within 7 days from the Tools Result Search function by searching in it using the result id.

This database also offers a gene function annotation search tool with a user-friendly graphic interface, “Gene Annotation Search”, which was embedded in the NHCCDB, based on *B. campestris* NHCC001 gene function annotation results. This functional unit is contained in the “Search” module on the main navigation bar. *B. campestris* NHCC001 gene function annotation results can be searched for using *B. campestris* NHCC001 gene IDs, GO and KEGG orthology identifiers, KOG, Pfam, SwissProt, TrEMBL, NR and NT domains. Here, we used the Pfam domain (WRKY) as an example to show the gene function annotation search results. By searching with WRKY as a query on the gene function annotation search page, 184 proteins in *B. campestris* NHCC001 are displayed (Figure 6b).

The SSR search function is used to search *B. campestris* NHCC001 simple sequence repeats. This functional unit is contained in the “Search” module on the main navigation bar. Simple sequence repeats can be searched using *B. campestris* NHCC001 gene IDs, SSRs, and SSR type. Here, we use SSR (CCG)6 as an example to show SSR search results. By searching using (CCG)6 as a query on the SSR search page, seven SSRs in *B. campestris* NHCC001 (BraC01g001690.1, BraC01g013590.1, BraC01g044420.1, BraC01g047960.1, BraC03g046720.1, BraC03g049520.1, and BraC08g030660.1) are retrieved (Figure 6c).

The NHCCDB database provides the “Synteny Gene Search” function to search syntenic genes between *B. campestris* NHCC001 and *A. thaliana* according to their necessity. This functional unit is contained in the “Search” module, and syntenic genes can be searched using *B. campestris* NHCC001 and *A. thaliana* gene IDs. We used the *B. campestris* NHCC001 gene *BraC01g032500.1* as an example to show syntenic genes in related species. By searching using *BraC01g032500.1* as a query on the Synteny Gene Search page, one syntenic gene in *A. thaliana* (*AT1G60990.1*) was retrieved (Figure 6d).

The NHCCDB database also provides the literature search function to search the desired literature according to need. Users need to enter the keywords in the text box provided for the search, and the results page will display the NCBI PubMed link to the article on submitting the task.

### 3.11. Download

The “Download” module has three divisions, namely, the Genome, SSR, and Demo Data. The Genome division provides the data of 17 genomes belonging to seven species for the users. The data from this division contain genome assembly, coding sequences, protein sequence, and GFF files. Additionally, the database of the genome division of the “Download” module provides eggNOG, Pfam, Swiss-Prot, and NR annotations to download. Moreover, GO and KEGG enrichment function datasets are available in this division. The SSR module contains 127,347 SSR sequences, from which 382,041 pairs of primers were designed. The Demo Data module displays eight bioplot functions and analyses performed by applying nine different tools to the example data.

### 3.12. Submit

The users of NHCCDB are encouraged to share their data with the research community. A Submit tool was embedded in NHCCDB. It collects not only data from genome, transcriptome, epigenome, and microarray but also functional data such as metabolome and proteome. With a user-friendly interface and simplified submission process, NHCCDB makes data submission and deposition very easy. Users can directly submit their data using the Submit function, and the users are required to provide their contact information. We also welcome suggestions from scientists all over the world to improve NHCCDB. If you have any questions during the submission process, please contact us.

### 3.13. Home Module

The “Home” module includes six different controls such as “About Us”, “Contact Us”, “FAQs” (Frequently Asked Questions), “News”, “Recent updates”, and “User guide”. The focus is primarily on the introduction of FAQs and on how to use this database.

## 4. Future Developments

In the future, the NHCCDB database will be updated regularly. Furthermore, the proteome sequences, metabolome, transcriptome, and phenomics data will be collected and stored in NHCCDB. Whenever high-quality genomic data of plants belonging to the Brassicaceae species is generated, the data will be collected and stored in NHCCDB, allowing the users to perform comparative genomic analyses.

In the Bioplot module, we provided eight types of biological plotting functions, which can be used to visualize multiple omics data. More plotting functions such as Sankey diagram plotting, radar diagram plotting, and chord diagram plotting will be added to NHCCDB, and users can use these functions to visualize the experimental data. With the development of new bioinformatics methods, more functions for multiple omics analysis will be developed and added to NHCCDB.

## 5. Conclusions

We developed the non-heading Chinese cabbage database (NHCCDB), a comprehensive and searchable database of the *B. campestris* NHCC001 genome. Numerous bioinformatics analysis functions have been incorporated into NHCCDB, allowing the users to submit, search, download, analyze, and visualize the *B. campestris* NHCC001 and its relative genomic data. NHCCDB will be updated regularly, and as more datasets become available, they will also be integrated. More analysis tools will also be added in the future. We also welcome suggestions from scientists worldwide to improve NHCCDB. We hope that the NHCCDB database will be a useful platform for non-heading Chinese cabbage and Brassicaceae species research and breeding.

## Data Availability

NHCCDB is freely available at http://tbir.njau.edu.cn/NhCCDbHubs/index.jsp (accessed on 11 January 2022). The website is optimized for Google Chrome, Internet Explorer, Mozilla Firefox, and Safari. All of the Illumina and Nanopore sequencing data are publically available at: https://www.tbirs.cn/NHCCDB/Genome.jsp (accessed on 13 January 2021).

## Supplementary Materials

The following supporting information can be downloaded at: https://www.mdpi.com/article/10.3390/plants11081005/s1, Table S1: The genomic data used in the present study. Figure S1: Screenshots of a genomic data page for *Brassica campestris* (syn. *Brassica rapa*) ssp. *chinensis* NHCC001 v1.0 Genome.
