# Supplementary material for: Non-Heading Chinese Cabbage Database: An Open-Access Platform for the Genomics of Brassica campestris (syn. Brassica rapa) ssp. chinensis
Source: Plants (Basel). 2022 Apr 7;11(8):1005. doi: 10.3390/plants11081005 (PMC9029197; doi:10.3390/plants11081005)
Supplement: Supplementary file 1 [file plants-11-01005-s001.zip › Supplementary Figure S1.pdf]

Brassica campestris (syn. Brassica rapa) ssp. chinensis (Cultivar NHCC001) v1.0 Genome

Brassica campestris (syn. Brassica rapa) ssp. chinensis (Cultivar NHCC001) v1.0 Genome

Box-plot | Venn-plot | QQ-plot | Manhattan-plot | Seqlogo-plot | Volcano-plot | Heatmap-plot | Corr-plot | Blast+ | SSR Finder | KEGG Enrichment | GO Enrichment | Synteny Finder | Motif Discovery | TF Annotation | Batch Query | Tools Result Search | Literature Search | Submit | JBrowse | Genome | Literature | Links | Primer Design

Overview:

**Family**	Brassicaceae
**Genus**	Brassica
**Species**	Brassica campestris (syn. Brassica rapa) ssp. chinensis
**Cultivar**	NHCC001
**Common Name**	Non-heading Chinese Cabbage

Feature Summary:

**Level**	Chromosome
**Chromosome**	2n=20
**Estimated genome size (Mb)**	477.76
**Genome sequence size (Mb)**	405.33
**Contig number**	602
**Contig N50 (Mb)**	2.83
**Contig max (Mb)**	22.49
**Scaffold number**	312
**Scaffold N50 (Mb)**	38.13
**GC content (%)**	37.13
**Gaps total length (kb)**	13,382
**Annotated gene number**	48,158

Reference Publication(s):

Figure S1. Screenshots of a genomic data page for *Brassica campestris* (syn. *Brassica rapa*) ssp. *chinensis* NHCC001 v1.0 Genome.
